# Natural source, bioactivity and synthesis of 3-Arylcoumarin derivatives

**DOI:** 10.1080/14756366.2022.2058499

**Published:** 2022-04-19

**Authors:** Qiang Zhang, Yu-hang Miao, Teng Liu, Yin-ling Yun, Xiao-ya Sun, Tao Yang, Jie Sun

**Affiliations:** a Institute of Materia Medica, Shandong First Medical University, Shandong Academy of Medical Sciences, Jinan, Shandong, China; b Department of Thoracic and Cardiovascular Surgery, First Affiliated Hospital of Chongqing Medical University, Chongqing, Chongqing, China

**Keywords:** Natural product sources, 3-arylcoumarin derivatives, bioactivity, synthesis

## Abstract

3-arylcoumarins with different pharmacological properties widely exist in a variety of natural plants. The extensive research on 3-arylcoumarins was attributed to its therapeutic and relatively easy isolation. Therefore, 3-arylcoumarins can be recognised as useful structures for the design of novel compounds with potential pharmacological interest, particularly in the fields of anti-inflammatory, anti-cancer, antioxidant, Monoamine oxidase (MAO) enzyme inhibition, etc. The current review highlights the biological activities, design, and chemical synthetic methods of 3-arylcoumarins derivatives as well as their important natural product sources.

## 1. Introduction

**Figure 1.**
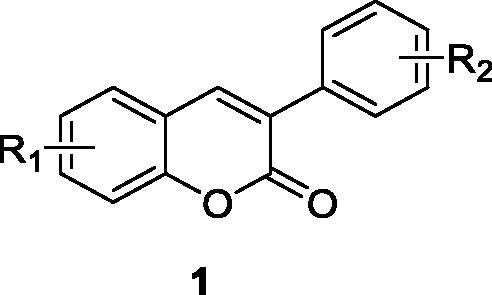
3-arylcoumarin skeleton.

3-Arylcoumarins refers to a sort of compound with a coumarin backbone and a 3-position aryl structural substitution^1^^,^^2^ (1, Figure 1). They are the secondary metabolites that have widespread metabolic functions in plants. They are widely distributed in fruits, flowers, seeds, vegetables and in both legumes and non-legume plants^3^. In the past decade, they have attracted increasing attention, and medicinal chemists have constructed a series of 3-arylcoumarin structures with diverse biological effects and studied their pharmacological activities such as antiviral^4^^,^^5^, antibacterial^6–8^, antimicrobial^9^, anti-inflammatories^10–13^, antiplatelet^14^, antioxidant activity^15–20^, antimitotic activity^21^^,^^22^, antidepressant^23^, and anti-Alzheimer’s disease^24^^,^^25^.

In recent years, the 3-phenylcoumarin skeleton has been studied in more detail in its antitumor activity^26–30^. It has been reported to inhibit cell growth and proliferation in various human cancer cell lines. Zhao et al. designed and synthesised a library of 3-arylcoumarin derivatives containing the structural features of KU-398 and silybin and identified them as novel inhibitors of Hsp90 protein. The compound showed the strongest anti-proliferative activity against SKBr3 cells and MCF-7 cells^31^. These indicate that some compounds have drug-like properties. In addition, the potential of 3-arylcoumarin derivatives was first known as potent, non-substrate, uncompetitive inhibitors of Theileria annulata enolase (TaENO)^32^. These compounds are the priority for the treatment of special disease.

## 2. Chemistry

### 2.1. Natural resource of 3-arylcoumarins

3-Arylcoumarin is the basic structure of a class of naturally occurring active compounds with an arylbenzopyrone as their basic backbone. This group of compounds has structural similarities to coumarin, flavone, isoflavone and the trans-stilbene backbone. They are also closely monitored as they are interrelated in the biosynthesis of plants. 3-arylcoumarins are a kind of compound that exists widely in natural products. Scientists have paid more attention to them, due to their extensive biological activities. 3-arylcoumarins are richly found in the plant species belonging to the family Campylotropis hirtella (Leguminosae)^33^, Cucumis bisexualis (Cucurbitaceae)^3^, Pterocarpus soyauxii^34^. Natural products containing 3-arylcoumarin compounds are mainly extracted and isolated from Glycyrrhiza uralensis Fisch., Glycyrrhiza glabra L. and Glycyrrhiza inflata Bat. (Licorice)^35–38^, Mucuna birdwoodiana (Leguminosae)^39^, Sphenostylis marginata ssp (Fabaceae)^40^ and Pongamiopsis pervilleana (Baill)^41^ (Table 1).

**Table 1.** Source of 3-arylcoumarin.

Sources	Compounds	Time	Ref.
*Glycyrrhizae radi*	**2–6**	1990,2000,2002,2017	^42–45^
*Erythrina indica*	**7,8**	2000	^46^
*Gnidia socotrana*	**9**	2002	^47^
*Trifolium repensL (Leguminosae)*	**10**	2003	^48^
*Dalbergia louveliiR. Viguier (Fabaceae)*	**11**	2003	^49^
*Derris scandens*	**12**	2004	^50^
*Glycyrrhiza glabra*	**13**	2005	^7^
*Glycyrrhiza uralensis*	**14-16**	2010	^51^
*Neorautaneniaspecies*	**17**	2006	^52^
*Asphodelus microcarpus*	**15,18**	2007	^53^
*Lotus polyphyllos*	**19**	2008	^54^
*Daphne giraldii NITSCHE*(Thymelaeaceae)	**20**	2008	^55^
*Campylotropis hirtella*	**21**	2008	^56^
*Mucuna Birdwodiana* Tutch	**22**	2010	^57^
*Selaginella tamariscina (Beauv .)*	**23**	2010	^58^
*Derris scandens (Fabaceae)*	**24,25**	2010, 2011	^59^ ^,^ ^60^
*Pongamiopsis perVilleana(Baill.)*	**26**	2010	^61^
*Tetramorium sp*	**27**	2012	^62^
*Millettia richardiana*	**28**	2013	^63^
*Pterocarpus santalinusL.*	**29**	2013	^34^
*Solanum indicumL. (Solanaceae)*	**30**	2014	^64^
*Pachyrhizus erosus(Fabaceae)*	**31**	2014	^65^
*Derris eriocarpa How (Tugancao)*	**32**	2015	^66^
*Ammi majus L. (Apiaceae)*	**33**	2017	^67^
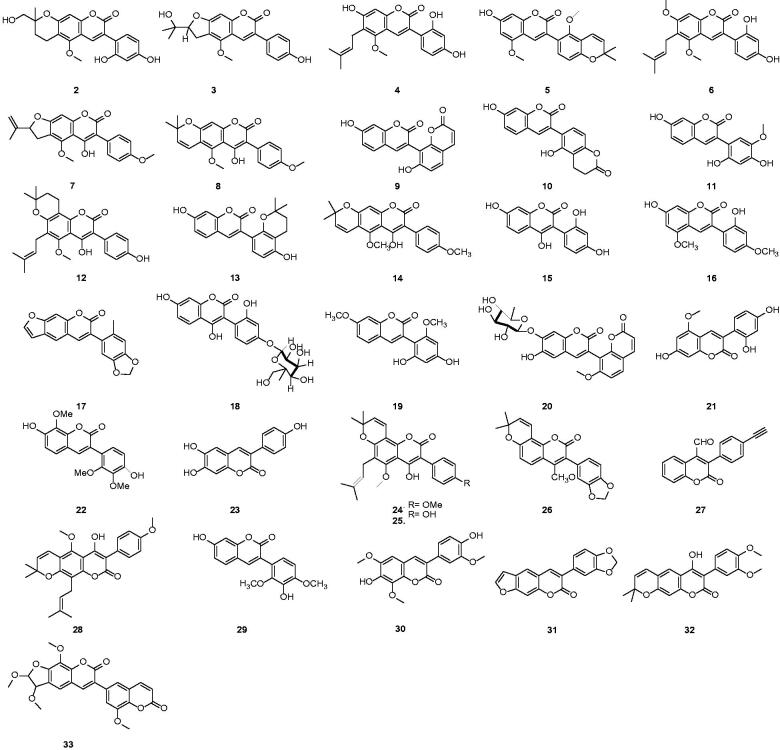			

### 2.2. Synthesis of coumarin fluorescent probes

With further research on the biological activity of 3-arylcoumarins, the study of its synthetic methods has become a focus in recent years. With the development of science, chemists are required to change the experimental process of high cost, high energy consumption and complex operation to the experimental conditions of low cost, low consumption and simple operation^16^. At present, the synthesis methods of 3-arylcoumarin compounds mainly include the closed-loop condensation reaction using phenol derivatives as raw material^13^^,^^68^, Palladium catalysed cross-coupling reaction of simple coumarins with aryl halide or aryl boric acid^69^, base promoted three-component coupling using arynes, malonates and DMF^70^. It also includes some classic name reactions, such as Pechmann condensation^71^, Perkin reaction^72–74^, Wittig reaction, Knoevengal condensation^71^.

#### 2.2.1. Perkin reaction

**Scheme 1.**
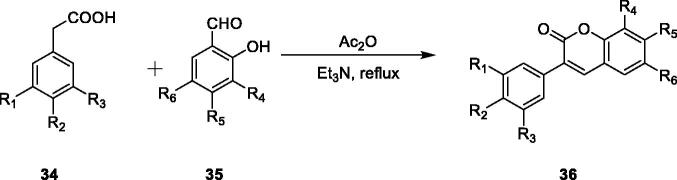
Perkin condensations between substituted phenyl acetic acids and *ortho*-hydroxylated benzal-dehydes selectively afford 3-arylcoumarins.

Perkin reaction is both the simplest and most direct method for preparing 3-arylcoumarin derivatives. According to Xiao et al.^75^ the Perkin condensation reaction of phenylacetic acid and o-hydroxybenzaldehyde was selected to selectively synthesise 3-arylcoumarin compounds, and these compounds were successfully synthesised by a simple and practical method (Scheme 1).

**Scheme 2.**
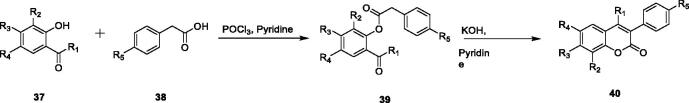
Methodology used for synthesis of 3-phenyl coumarins.

Taksande et al.^76^ reported a simple two-step synthesis method of 3-aryl-4-substituted coumarin derivatives. Firstly, the appropriate substituted 2-hydroxycarbonyl compounds were selected for acetylation with aryl acetic acid. Then, aryl acetoxy ester and base (pulverised potassium hydroxide) in pyridine carry out Dickmann cyclisation reaction to obtain the corresponding 3-aryl-4-substituted coumarins. In the second step, the yield of the ester reacting with potassium carbonate in dimethylformamide (DMF) or triethyl amine is not as high as that in pulverised potassium hydroxide and pyridine. The method has the advantages of cheap raw materials, quick reaction at room temperature, and no metal catalysis (Scheme 2).

#### 2.2.2. Pechmann reaction

**Scheme 3.**
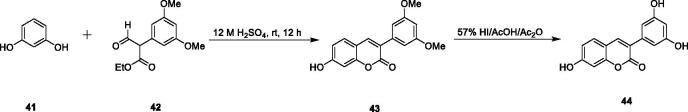
Methodology used for synthesis of compound **46**.

Pechmann reactions can replace Perkin and Knoevenagal reactions because the last is limited in the synthesis of coumarins with certain substituted salicylaldehydes. Vilar et al.^77^ used Pechmann's reaction to substitute a series of phenol and β-keto esters as raw materials and reacted under the action of concentrated sulphuric acid to obtain a series of 3-arylcoumarin derivatives. Wang^78^ summarised some new catalysts that can improve the yield of Pechmann reaction, such as Well-Dawson heteropoly acid, solid super acid, phenylsulfonic acid-functional molecular sieve. Such catalysts can shorten the reaction time and promote the reaction conditions (Scheme 3).

#### 2.2.3. Other method

**Scheme 4.**
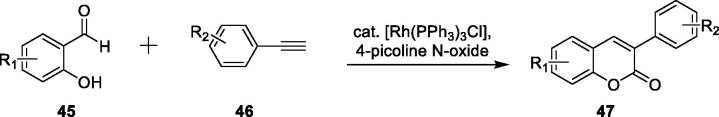
Rhodium-catalysed annulations of aldehydes with alkynes.

The different catalysts can lead to distinct products in the same reaction. Different metal catalysts were used to control the selectivity of the reaction with the same starting materials to build different natural product skeletons. Zeng^79^ group can effectively obtain coumarin skeletons by ring reaction of terminal alkyne and salicylaldehyde with rhodium catalyst instead of gold. It was also found that all kinds of substituents on arylacetylene have little effect on the reaction results. In all situations, different 3-aryl coumarins could be obtained by this method in significant yields (Scheme 4).

**Scheme 5.**
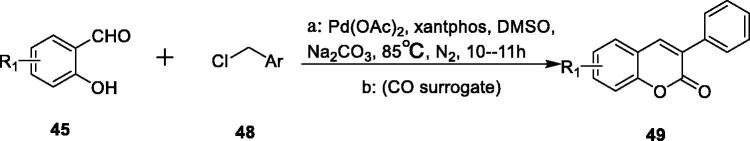
Pd-catalysed carbonylative synthesis of 3-arylcoumarins.

Yadav et al.^80^ reported a simple and efficient method. Under palladium-catalysed conditions, salicylaldehyde and benzyl chloride undergo carbonylation cyclisation to obtain higher yields of 3-arylcoumarin compounds. This method uses inexpensive, readily available and easily handled N-formyl saccharin to replace toxic carbon monoxide as the CO source, thereby reducing the toxicity of the reaction (Scheme 5).

**Scheme 6.**
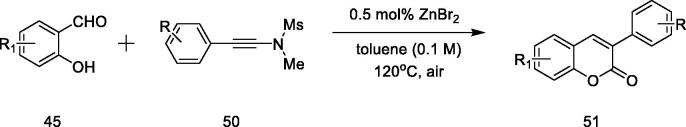
Zn-catalysed carbonylative synthesis of 3-arylcoumarins.

Yoo et al.^81^ published a method for the synthesis of 3-aryl coumarins with Zn(II) metal as catalyst and ynamides and salicylaldehyde as substrates. The amidosulfonates were successfully recovered in this process, providing an effective traceless guide group for the bonding process of the highly regioselective compounds. The method has the advantages of high functional group capacity, a wide range of substrates, simple reaction, high yield, the high recovery rate of sulphonamides and low sulpha drug loading, which is an economical and environmentally friendly process (Scheme 6).

The application of transition metal complexes as catalysts for the preparation of various 3-aryl coumarins has made great progress but still suffers from certain drawbacks. For example, multi-step reaction processes with demanding reaction conditions and require precious metal catalysts.

**Scheme 7.**
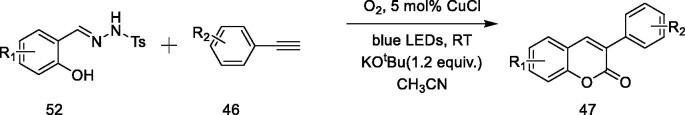
Pd-catalysed carbonylative synthesis of 3-arylcoumarins.

In 2019, Ragupathi et al.^82^ presented the first sustainable, intuitive, highly regioselective, visible-light-driven copper-catalysed aerobic oxidative cascade cyclisation reaction with terminal alkynes to prepare 3-aromatic coumarins at room temperature. This method is suitable for a wide range of substrates and gives a good yield of product (Scheme 7).

**Scheme 8.**
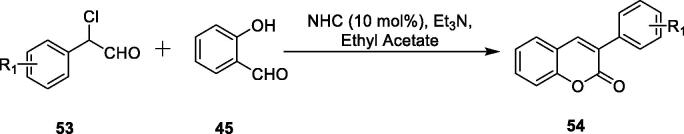
N-heterocyclic carbene catalyses the reaction to obtain 3-arylcoumarin efficiently.

Jiang et al.^83^ explored a reaction method for obtaining 3-arylcoumarin with high yield under mild conditions catalysed by N-heterocyclic carbene. Under a very simple experimental operation, the target product can be successfully obtained by using 2-chloro-2-arylacetaldehyde and o-hydroxybenzaldehyde as reaction substrate (Scheme 8).

#### 2.2.4. Coumarin substitution method

Coumarin substitution methods are available as follows. Direct arylation of coumarins in the presence of metallopalladium catalysis, using inexpensive sulphonyl or sulphite, constructs biologically significant 3-arylcoumarins and precludes prefunctionalization of coumarins^84^, Versatile palladium-catalysed C–H olefination of (hetero)arenes at room temperature^85^. The regioselective arylation of coumarins with phenylhydrazine^86^^,^^87^ or phenylboronic acid^88^^,^^89^ under transition metal-free conditions gives 3-aryl coumarins.

**Scheme 9.**
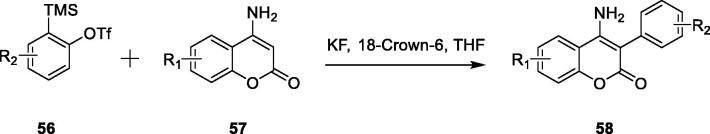
Synthesis of 4-amino-3-arylcoumarins from unsymmetrical benzyne precursors.

A highly efficient, mild and transition metal-free 4-aminocoumarin α-arylation strategy was developed. This method used 4-aminocoumarin and aromatics as raw materials to form a C (Sp^2^)-C (Sp^2^) bond to directly synthesise 4-amino-3-arylcoumarin derivatives. In the absence of a metal catalyst, the reaction was completed in one step. The product has better functional group compatibility (including halogen substituents) and higher yield (Scheme 9)^90^.

## 3. Activity of 3-arylcoumarin derivatives

Many 3-arylcoumarins compounds not only can be isolated from plants but also can be synthetised by different methods. The literature indicates that 3-arylcoumarin compounds have a wide range of therapeutic potentials^91^. The pharmacological activities, as well as therapeutic applications of 3-arylcoumarins, will change depending upon the pattern of substitution at different positions of the ring. Due to the various activities of 3-arylcoumarin, it gradually becomes the focus. In this review, the biological activities of 3-arylcoumarins and their derivatives are classified and described (Table 2).

**Table 2.** 3-Arylcoumarins and their activities in recent years.

Structure	Biological activity	Ref.
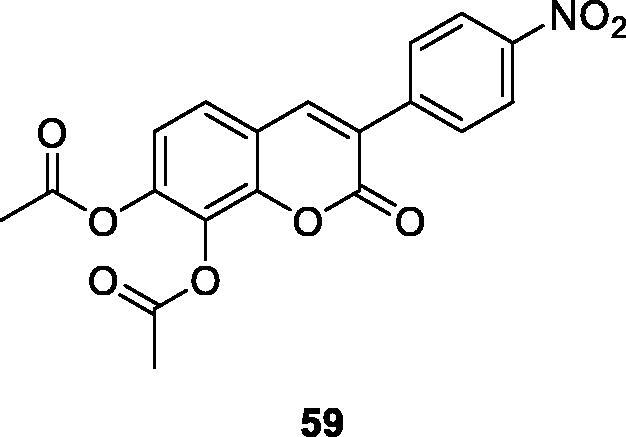	Anti-tumour Cytotoxicity against A549, MDAMB-231 and PC3 cancer cell lines.	^97^
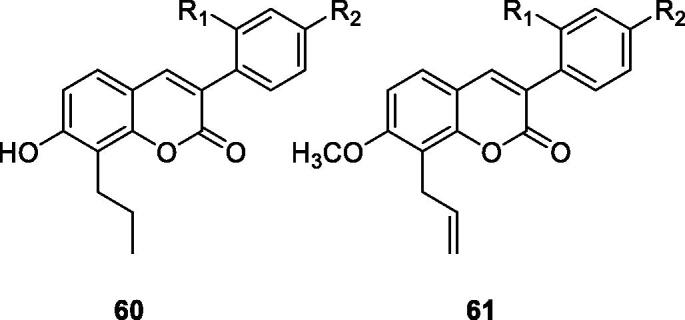	a: R_1_=R_2_=OCH_3_ b: R_1_=C_l_, R_2_=H Anti-cancer activity Anti-cancer activities on SK-Hep-1, HepG2, HeLa and SGC7901 cell lines	^98^
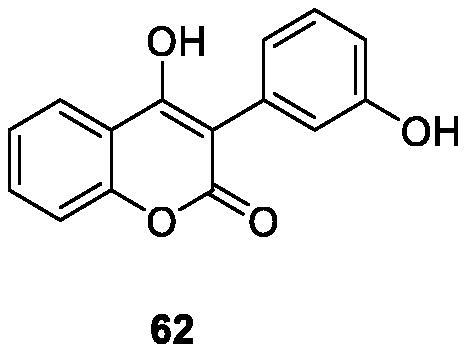	Anti-proliferation activity of MCF-7 cells	^99^
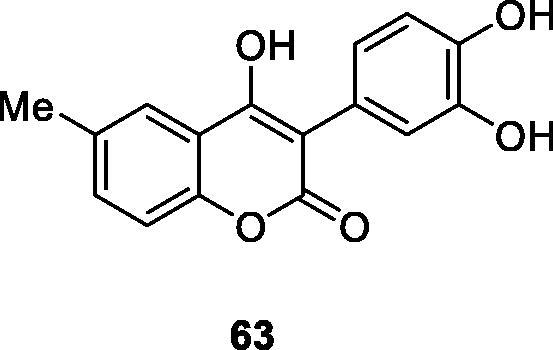	Anti-proliferation activity most effective in HL-60 cells	^99^
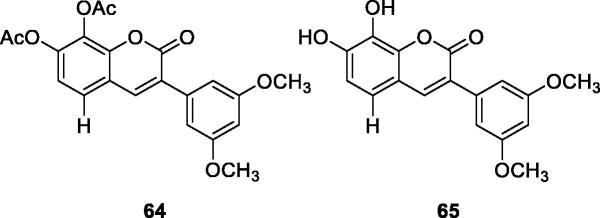	Anti-tumour As a potential antitumor drug	^100^
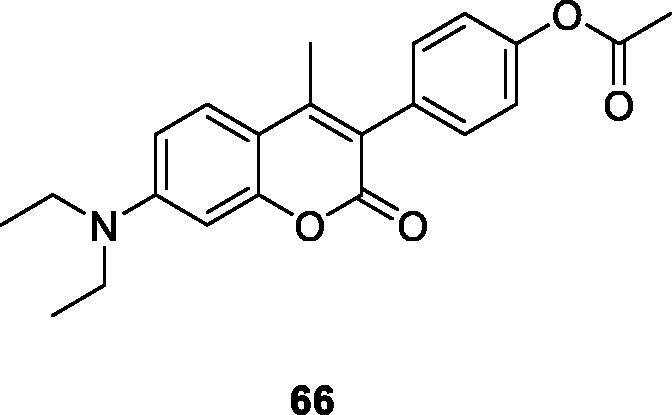	Anti-cancer activity Multifunctional anti-tumour agent	^94^
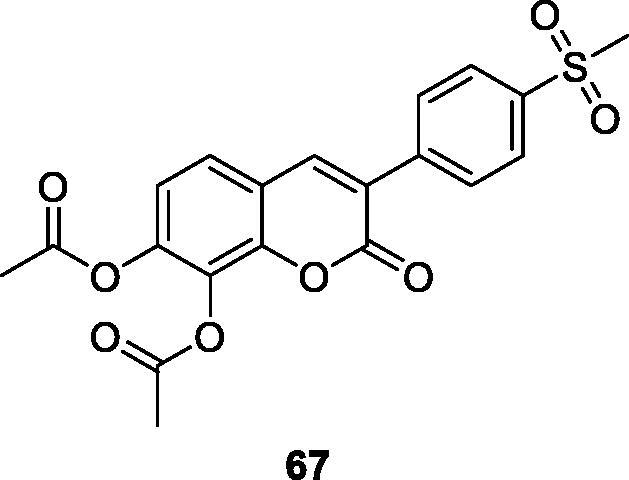	Anti-tumour cytotoxic activity	^101^
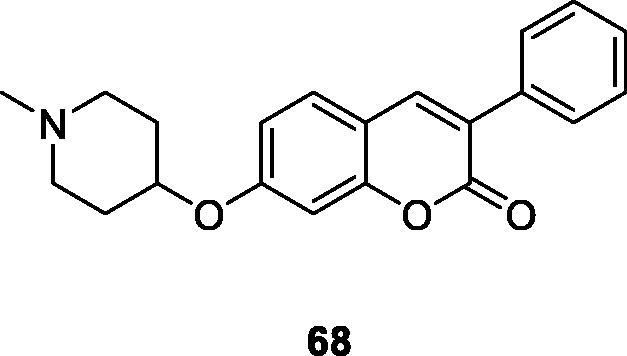	Anti-proliferation activity	^31^
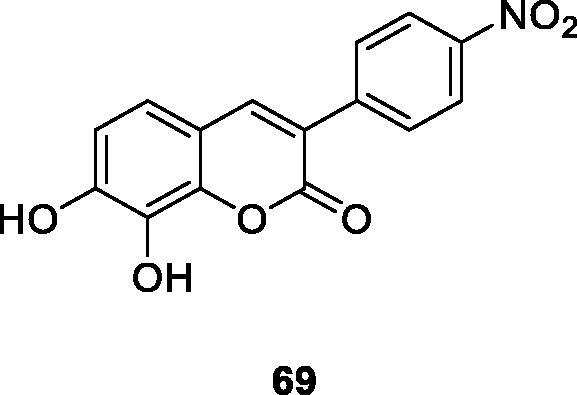	Anti-tumour Cytotoxic effects on human liver cancer (HepG2), prostate cancer (LNCaP) and pancreatic cancer (BxPC3) cell lines	^102^
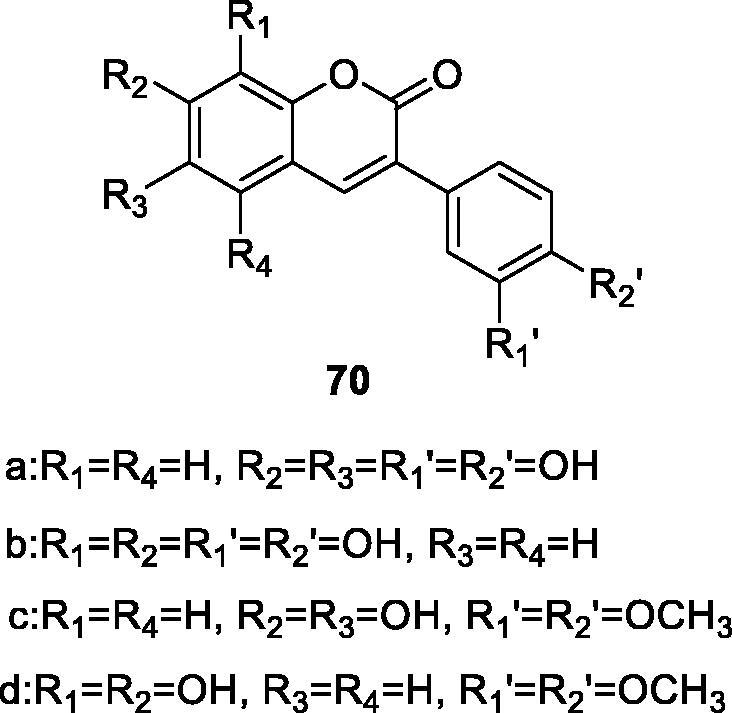	1. 28 Anti-tumour Inhibitory effect on human placental glutathione S-transferase (GSTP1-1)	^103^
	2. 28a: Antioxidant activity	^18^
	Anti-inflammatory	^43^
	Antibacterial activity	^6^
	28b: Antioxidant activity	^20^
	Antibacterial activity	^6^
	Theileria annulata enolase (TaENO) inhibitors	^32^
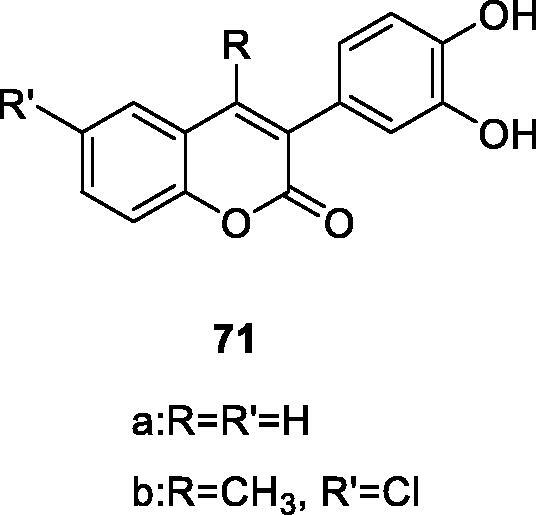	Antioxidant activity Potential antioxidant	^105^
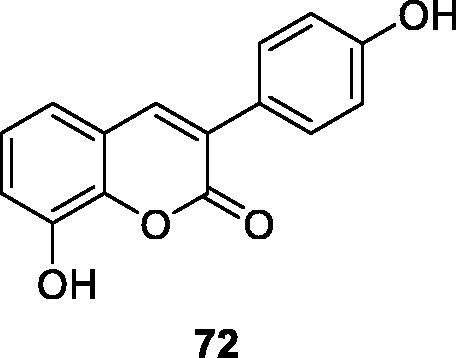	Antioxidant activity The ability to scavenge free radicals is 100%	^104^
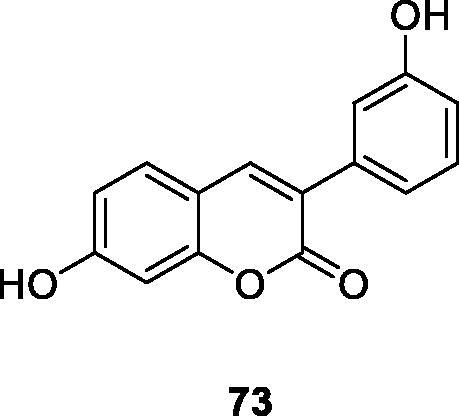	Antioxidant activity As potential candidate compounds	^106^
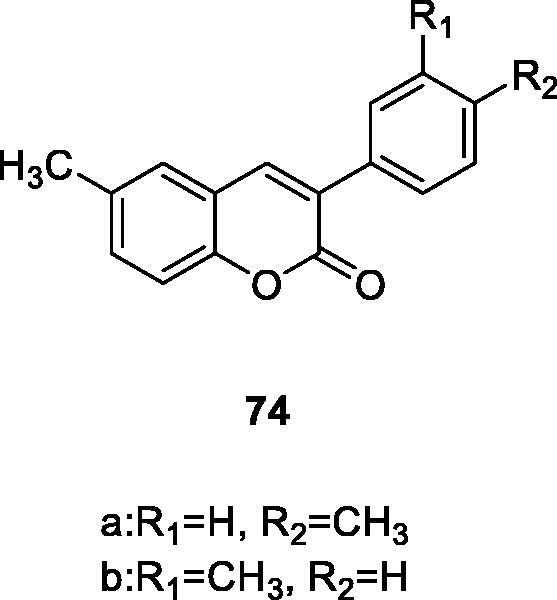	Selective Monoamine Oxidase B Inhibitors	^107^
	74a: Adenosine affinity	^125^
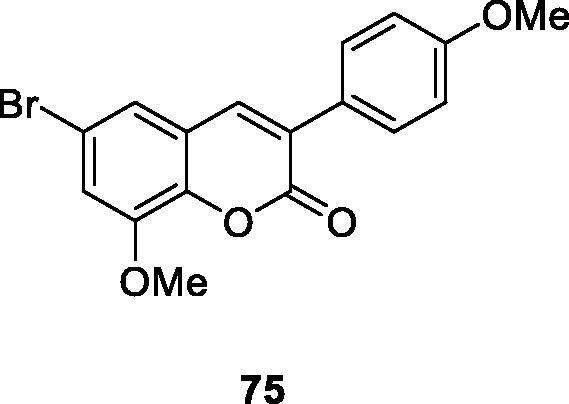	MAO-B Inhibitors	^108^
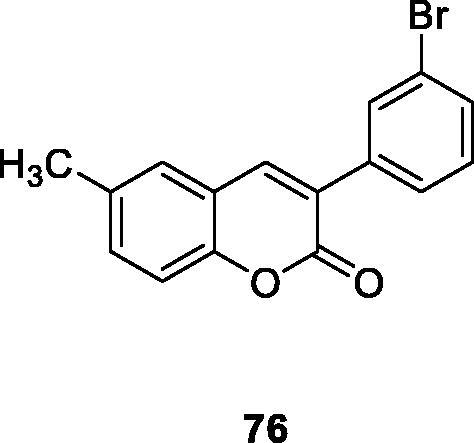	MAO-B Inhibitors Most active compound	^109^
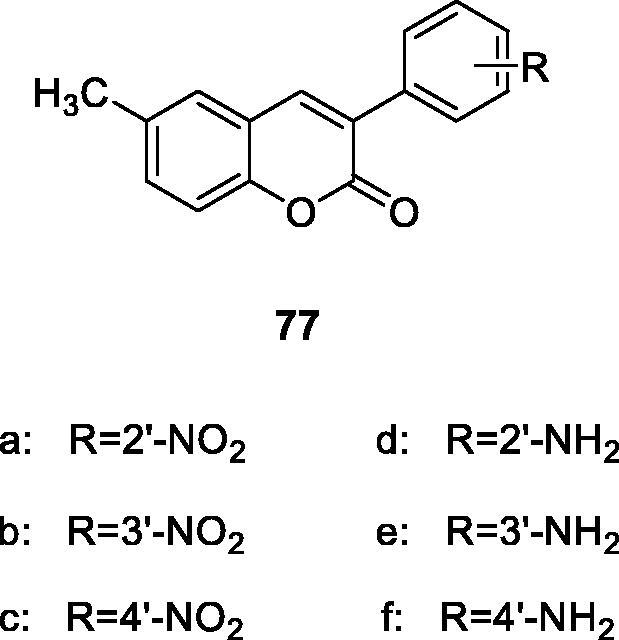	MAO-B Inhibitors	^110^
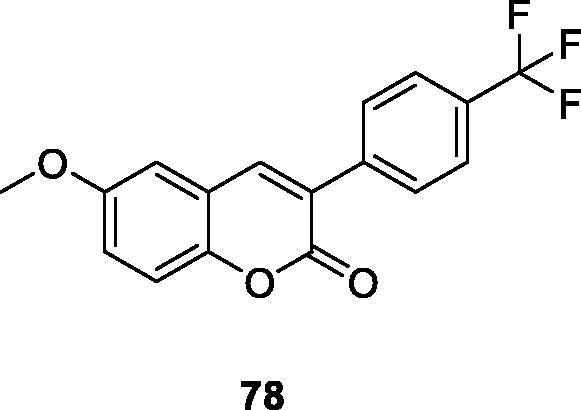	MAO-B Inhibitors An ideal framework for constructing effective small molecule MAO-B inhibitors	^111^
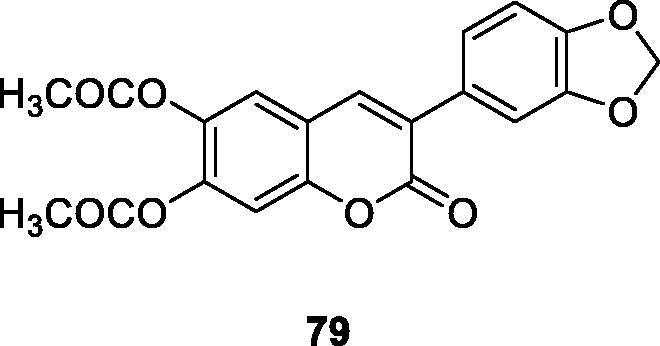		
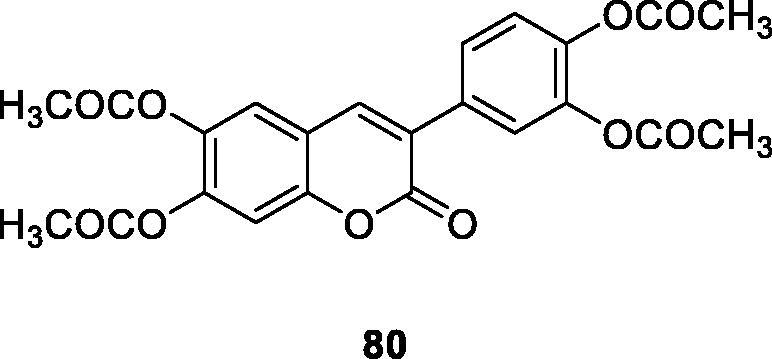	Anti-inflammatory	^11^
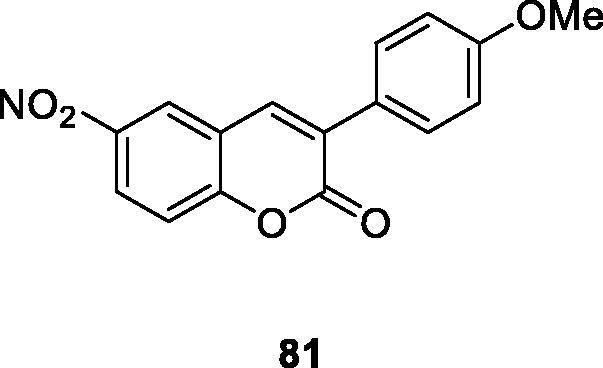	Anti-inflammatory XO non-competitive inhibitor Anti-PD activity	^12^ ^ ^ ^114^
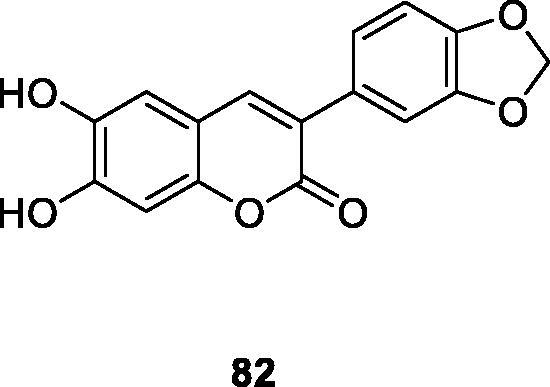	Anti-inflammatory Reduce neutrophil oxygen free radicals and total ROS levels	^43^
	2.Down-regulating neutrophil function	^3^
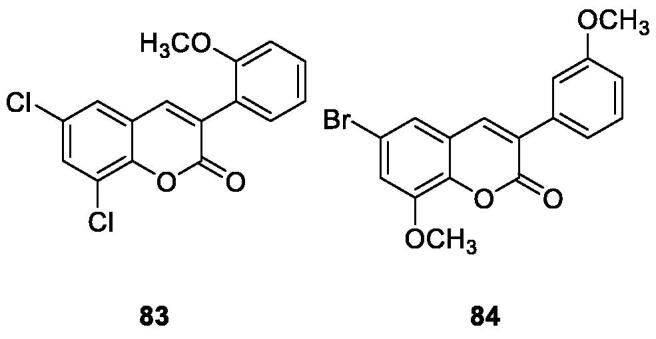	Anti-inflammatory As a potential anti-inflammatory lead compound	^13^
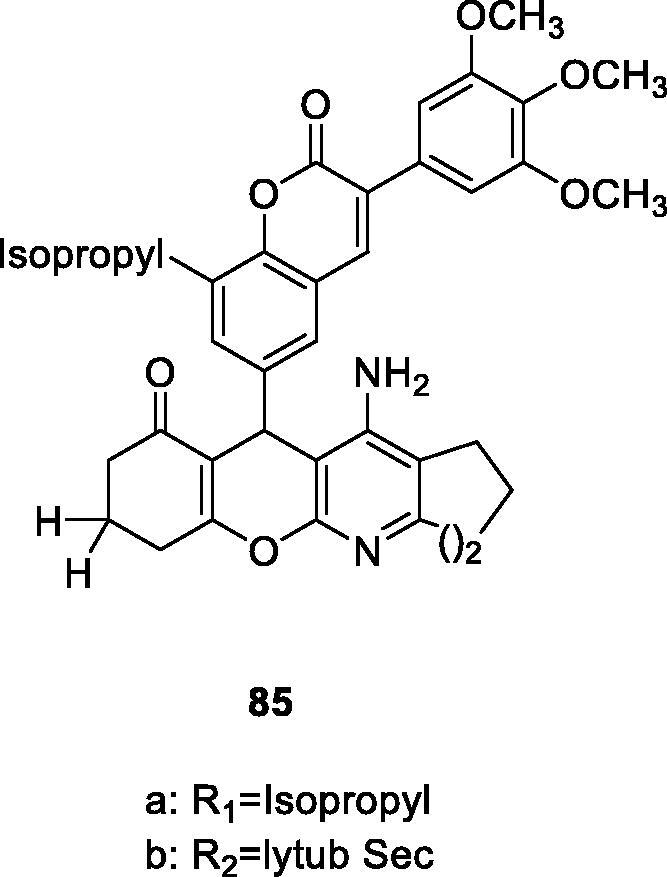	Anti-PD activity As a structure to treat Parkinson's disease	^115^
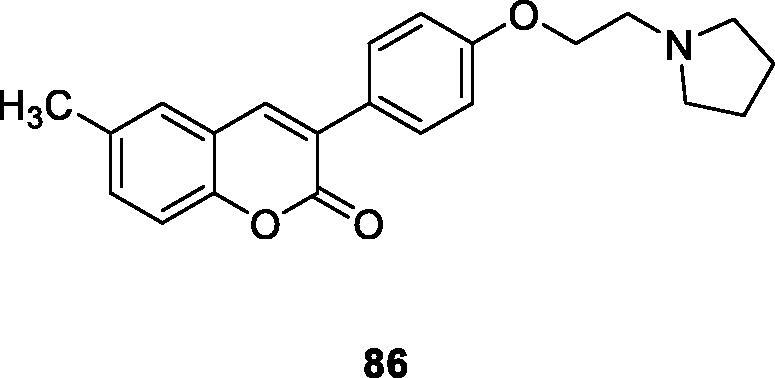	Anti-AD activity As a selective AChE / MAO-B double inhibitor	^117^
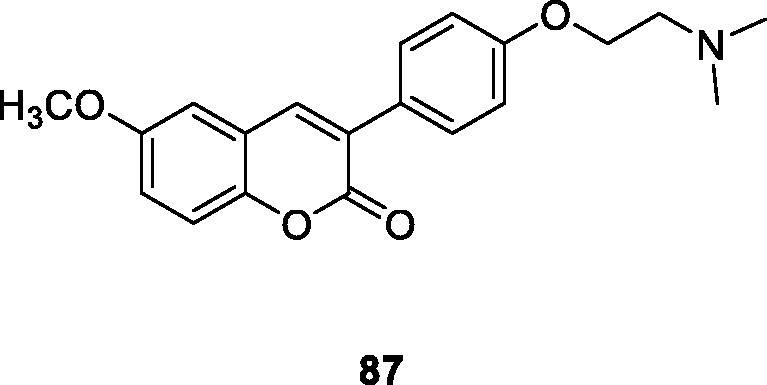	Anti-AD activity	^116^
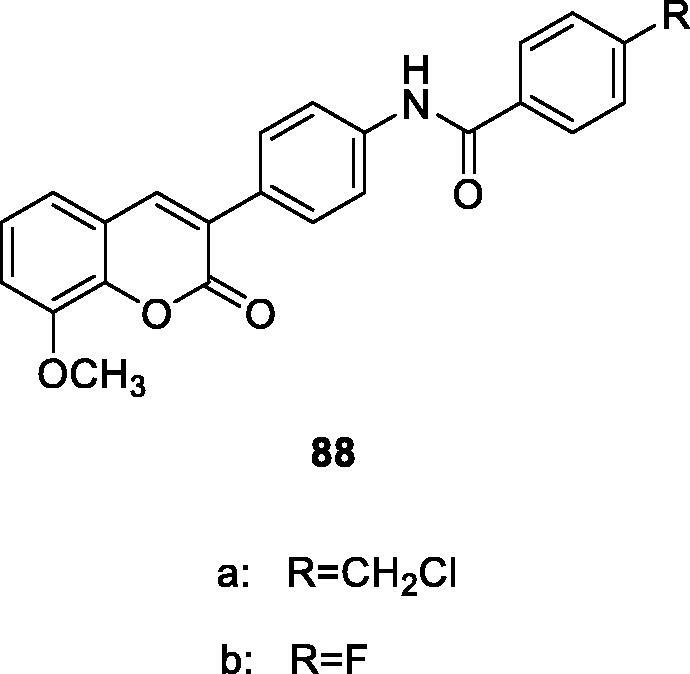	Anti-diabetic activity	^120^
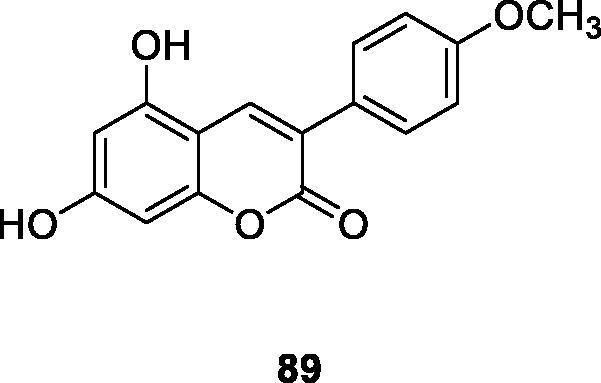	Antibacterial activity Inhibitory activity against S. aureus	^119^
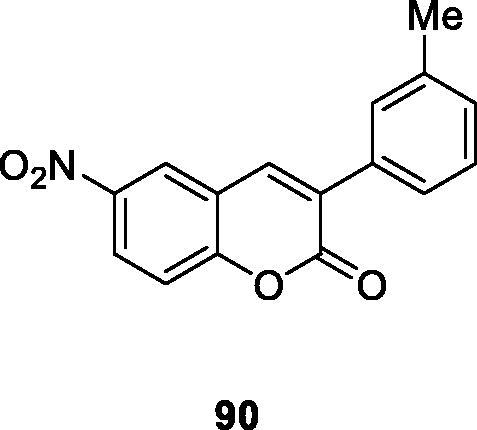	Antibacterial activity As potential candidate compounds	^122^
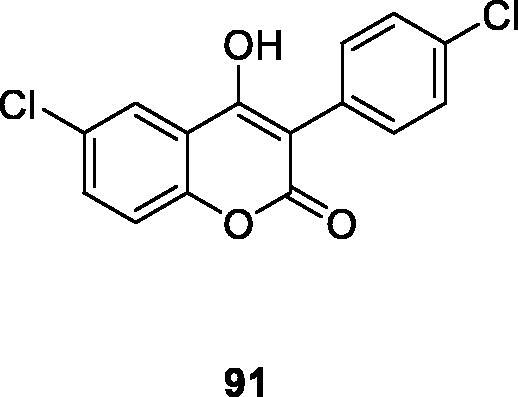	Tyrosinase inhibitory activity	^19^ ^ ^ ^124^
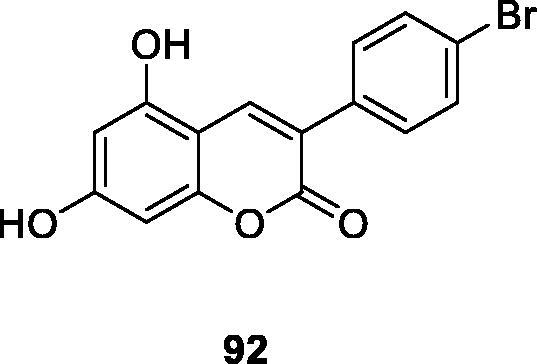	Tyrosinase inhibitory activity	^123^
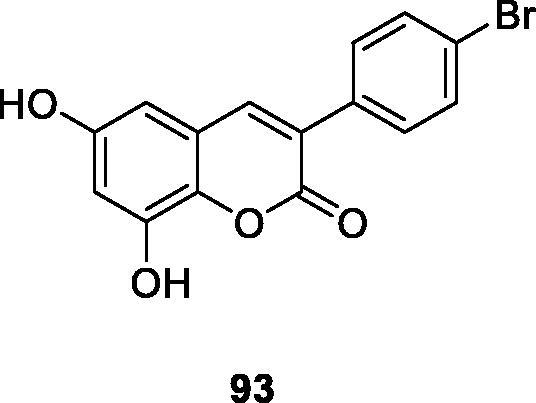	Anti-insect activity	^20^
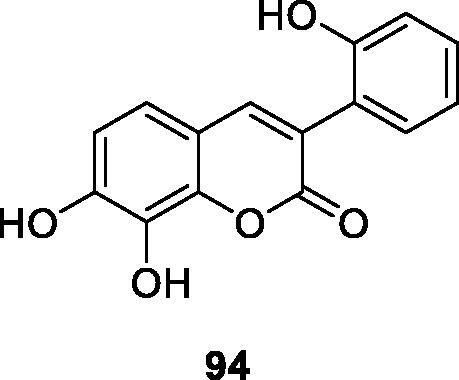	Adenosine affinity	^126^
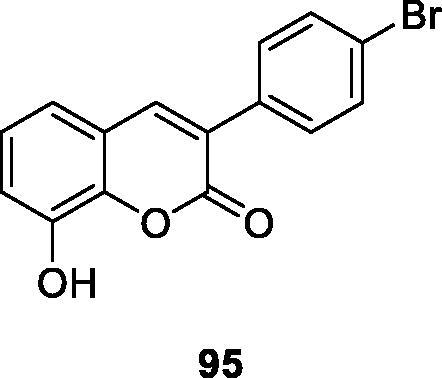	Anti-platelet activity	^14^
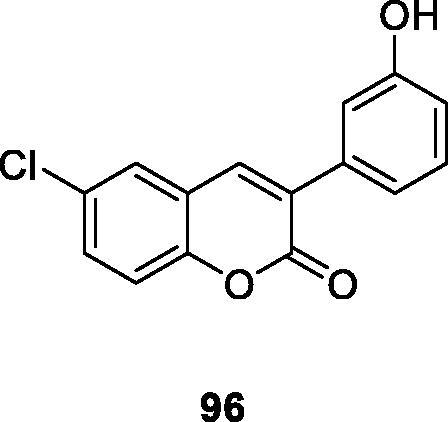	Mast cell degranulation inhibitor As new antiallergic drugs	^127^
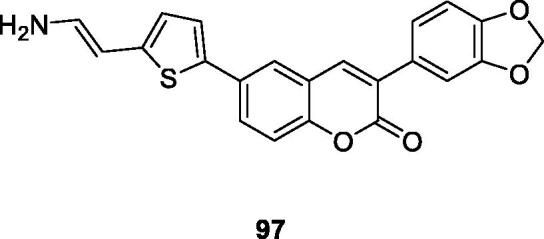		

### 3.1. Anti-tumour activity and cytotoxicity

Malignant tumours are expected to be the leading cause of death in lifespan in most countries in the 21^st^ century^92^. Therefore, there is an urgent need to find effective anti-cancer drugs and treatments.3-Arylcoumarins are derived from natural products with low toxicity and have achieved good therapeutic effects.

Research on its antitumor activity has never stopped over the past decade years. Biological studies of 3-arylcoumarins have revealed that these compounds act as an anti-cancer and myriad pathway. Targets include kinase inhibition, cell cycle arrest, angiogenesis inhibition, heat shock protein inhibition, antimitotic activity, etc^28^^,^^93^. 48For example, Musa et al. discovered that 3-arylcoumarin compounds can not only inhibit human lung cancer cells (A549) in a concentration-dependent manner by arresting cells in the S phase^94^^,^^95^, but also induces the apoptosis by increasing the expression of pro-apoptotic Bax and decreasing that of anti-apoptotic Bcl-2^96^. The subject then combined a variety of 3-arylcoumarin derivatives to study their anticancer activity against various cell lines, elucidate the mechanism of action, and discover a series of meaningful lead compounds for the treatment of cancer.

Musiliyu et al.^97^ had interested in synthesis and biological evaluation of 3-arylcoumarins as anti-cancer agents. This group had synthesised a series of 3–(4-nitrophenyl)-aryl coumarin derivatives and evaluated their *in vitro* cytotoxic effect on A549, MDAMB-231 and PC3 cancer cell lines. The results of this study indicated that the presence of diacetoxy group at the carbon 7, 8 positions can enhance cytotoxic activity in various cancer cell lines. In vitro cytotoxic effect of those compounds was evaluated at different concentrations. Compound **59** in A549 (CC_50_ =12.2 µM), MDA-MD-231 (CC_50_ = 27.6 µM) and PC3 (CC_50_ = 18.2 µM) cancer cell lines, while other compounds did not show cytotoxic effect (CC_50_ > 100 µM).

Liu et al.^98^ designed and synthesised a series of 3-arylcoumarin derivatives. The *in vitro* cytotoxic activity was evaluated on SK-Hep-1, HepG2, HeLa and SGC7901 cell lines were measured by MTT method. The results displayed that compounds **60a**, **60b**, **61a** and **61b** had significant selective anti-proliferative activity on different types of cancer cell lines *in vitro*. In particular, compound **61a** revealed the strongest activity, and its IC_50_ value of 6.75 µM to against HeLa cell line. The key to increasing the anti-proliferative activity of C8-allyl and 7-methoxy.

Very simple 4-hydroxycoumarin was found to cause selective cytoskeletal disintegration in melanoma cells without affecting non-tumour fibroblasts. Therefore, Serra et al.^99^ investigated the existence of different substituents on C-6 and the effect of introducing hydroxyl groups into the 3-aryl-4-hydroxycoumarin ring at different positions to inhibit its proliferative activity. Among all coumarins synthesised, only compound **62**, which has m-hydroxy group on 3-aryl ring, is more effective than simple coumarin in anti-proliferation activity of MCF-7 cells and inferior to reference compound tamoxifen. Compound **63** has been proved to be the most effective in HL-60 cells, but it is also far lower than the reference compound tamoxifen.

Xiao et al.^100^ designed and synthesised a series of 3-arylcoumarin compounds known as coumarin-stilbene hybrid compounds in high yield and evaluated them as potential antitumor agents. Among them, the IC_50_ value of compound **64** on KB cells was 5.18 µM, which was the most active compound. The inhibitory effects of compounds **64** and **65** on MCF-7/ADR cell lines were significantly higher than normal MCF-7 cell lines, with IC_50_ values of 11.94 and 11.11 µM, respectively. In summary, 3-arylcoumarin compounds containing 7,8-dihydroxy or 7,8-diacetoxy may have strong cytotoxicity *in vitro* and can be further studied.

Musa et al.^94^ believed that one of the current methods to improve cancer treatment is protein acetylation. Studies demonstrated that the presence of TAase in the liver catalyses the transfer of acetyl groups from 7,8-diacetoxy-4-methylcoumarin (DAMC) to certain receptor proteins, thereby regulating their catalytic properties. At the same time, DAMC also has antitumor activity. These studies strongly support the potential antitumor applications of coumarin analogs. Therefore, the research group reported new acetoxycoumarins and their cytotoxic activity *in vitro*. The results showed that compound **66** had cytotoxic activity on A549 lung cancer, CRL1439 normal liver cells and CRL1548 liver cancer, LD50 value of A549, CRL1439 and CRL1548 cell lines 48.1, 45.1 and 49.6 µM, respectively. The compound can induce cell cycle arrest of A549 lung cancer cells and CRL1548 liver cancer cells in different phases, which can be used as a lead compound for the development of potential anti-tumour in the future.

Studies manifested that the presence of 7,8-diacetoxy on the 3-arylcoumarin molecule enhances drug activity, such as antioxidant, anti-cancer, and scavenging free radicals. Therefore, a series of 7,8-diacetoxy-3-arylcoumarin derivatives were synthesised and evaluated for the *in vitro* cytotoxic activity of PC-3 and MDA-MB-231 cancer cells. Structure-activity relationship (SAR) studies clearly show that the cytotoxic activity of 7,8-diacetoxy-3-arylcoumarins depends on the cell line and the type of substituted aryl group at the C-3 position. The results indicated that compound **67** had the strongest cytotoxic activity on PC-3 cell lines (CC_50_ = 26.43 µM). However, it has no cytotoxic activity on non-cancerous human prostate cell lines (WPE1-NA22) (CC_50_ > 100 µM), so it can be concluded that the compound has selective cytotoxicity. In addition, compared with the reference drug Tamoxifen (TAM), the toxic activity on PC-3 and MDA-MB-231 cancer cell lines was similar or slightly lower^101^.

Zhao et al.^31^ designed and synthesised a library of 3-arylcoumarin derivatives, which retained the coumarins ring, and also contained structural features of KU-398 and silybin. KU-398 and silybin are Hsp90 (90 kDa heat shock protein) inhibitors. The newly synthesised compound was evaluated for the activity of two breast cancer cell lines and found that compound **68** showed the strongest antiproliferative activity, with an IC_50_ value of 0.98 ± 0.01 µM against SKBr3 cells and 0.81 ± 0.02 µM against MCF-7 cells. Western blot showed that compound **68** can bind to the C-terminus of Hsp90 and inhibit the increase of Hsp90 levels, so it can resist cancer cell lines proliferation.

Musa et al.^102^ reported that the synthesis of a series of 3-arylcoumarin derivatives has *in vitro* cytotoxic effects on human liver cancer (HepG2), prostate cancer (LNCaP) and pancreatic cancer (BxPC3) cell lines. The results showed that compound **69**, 7,8-dihydroxy-3–(4-nitrophenyl)-coumarin had the strongest effect on HepG2 cell lines. And the mechanism of action showed that compound **69** blocks HepG2 cells and the S phase of the cell cycle, leading to reactive oxygen species (ROS)-independent cell death. All in all, compound **69** can be a valuable lead compound for the treatment of liver cancer.

Alparslan et al.^103^ designed and synthesised several substituted 3-arylcoumarins and study their inhibitory effect on human placental glutathione S-transferase (GSTP1-1). Glutathione S-transferases (GSTs) can catalyse the binding of multiple electrophilic compounds to glutathione. There is evidence that the high expression of GSTs is the main reason for the resistance of tumour cells to chemotherapy drugs. So, acetylene acid, a potent GST inhibitor, was used as a positive control to compare the performance of the synthesised compounds. Among them, compounds **70a** and **70b** with four hydroxyl substituents had the highest inhibitory activity on human placental GST enzymes, with IC_50_ values of 13.5 and 17.47 µM, respectively. The inhibitory activities of 6,7-dihydroxycoumarin derivatives **70a** and **70c** were significantly higher than those of 7,8-dihydroxycoumarin derivatives **70b** and **70d**. It can be seen that 6,7-dihydroxy-3-arylcoumarin is expected to become a new GST inhibitor designed as an adjuvant for the treatment of tumours to overcome the multidrug resistance of tumour cells caused by GST overexpression.

### 3.2. Antioxidant activity

Normal metabolism in the body can produce reactive oxygen species. If ROS is produced in large quantities in the body or the balance between generation and natural elimination of ROS is broken, oxidative stress occurs. It will induce oxidative damage in biomolecules, such as proteins, lipids, nucleic acids and biofilms. Diseases indirectly related to oxidative stress, such as cancer, cardiovascular disease and chronic diseases such as neurodegeneration^20^. So it is important that antioxidants in protecting organisms from oxidative disorders, and they can reduce or prevent the oxidation process through different mechanisms. Some of the 3-aryl coumarin derivatives containing hydroxyl groups have good antioxidant activity. The structures are shown in the table above^20^^,^^104^^,^^105^.

### 3.3. Selective monoamine oxidase B inhibitors

Monoamine oxidase is a FAD-dependent enzyme, existing in the outer mitochondrial membranes of glial cells, nerve cells, and other mammalian cells. These enzymes can control the levels of biogenic amines in the brain and peripheral tissues by catalysing the oxidative deamination of food amines and neurotransmitters^19^. It can be divided into two isoforms, MAO-A and MAO-B, based on the amino acid sequence, three-dimensional structure, tissue distribution and inhibition selectivity of the enzyme^106^. Because of their different affinity to different substrates, the two enzymes are involved in different pathological processes. The hMAO-A isoform has a higher affinity for serotonin and norepinephrine, while the hMAO-B isoform preferentially deaminates β-phenylethylamine and benzylamine^18^.

In the past ten years, Matos et al. have devoted themselves to the research of more effective and less toxic MAO-B selective inhibitors and synthesised a series of 3-arylcoumarin derivatives. A new 3-arylcoumarin skeleton with high MAO-B selectivity was found. In 2011, in order to search for the MAO inhibitory activity and selectivity, a series of new 6-substituted-3-arylcoumarins were synthesised, evaluated and compared. Most of the compounds studied showed higher affinity and selectivity for hMAO-B isozymes than the selegiline (IC_50_ = 0.0196 µM, reference MAO-B inhibitor), with an IC_50_ value in the appropriate range (from nanomoles to picomoles). The two most active compounds were coumarin derivatives **74a** (IC_50_ = 0.31 × 10^−3 ^µM) and **74b** (IC_50_ = 0.80 × 10^−3 ^µM), which had methyl groups at C6 and methyl or methoxy groups were found in the para-position of the 3-benzene ring, respectively^107^. SAR research found that when a coumarin mother nucleus had a phenyl group at the C3 position, a larger substituent at the C6 position will reduce the inhibitory activity on MAO-B. Moreover, the type and position of the substituent on the 3-aryl group play a vital role in activity and selectivity, and *meta* and *para* substitutions are the most favourable positions for the target activity.

To find the structural characteristics of 3-arylcoumarin compounds’ inhibitory activity and selectivity to human MAO. Matos et al.^108^ reported four 3-phenylcoumarins were reported, which were at the C6 and the C8 of the 3-arylcoumarin. A bromine atom and a methoxy/hydroxy substituent were introduced at the C6 or C8 position. Furthermore, the synthesised compounds were evaluated for MAO-A and B inhibitors. The results indicated that this method enhances the potency and selectivity of the described 3-phenylcoumarin compounds for MAO-B. In addition, the introduction of a substituent at the para-position of 3-phenyl may benefit from increasing the activity. When a substituent is a methoxy group (compound **75**), the inhibitory activity against MAO-B is the highest.

There are 25 kinds of 3-arylcoumarin derivatives that were synthesised by Perkin reaction, hydrolysis reaction and Williamson reaction effectively. And their ability to inhibit hAMO-A/B was evaluated. It can be observed from the experimental results that compound **76** was the most active compound in the series. The inhibitory activity on MAO-B is more than 140 times that of the positive control selegiline, which has obvious advantages in terms of selectivity and reversibility^109^.

Matos et al.^110^ reported a series of new amino and nitro 3-phenylcoumarin derivatives (compound **77**) were synthesised and evaluated as MAO-A/B inhibitors. The results showed that amino derivatives were more selective for MAO-B than nitro derivatives, and derivatives with *para* and *meta* substituents inhibit MAO-B activity more strongly than *ortho* substituents.

Sanna et al.^111^ reported a series of 3-phenylcoumarin derivatives were designed using a virtual combination chemistry method. The addition of methoxy, hydroxyl, acetoxy and methyl groups to the 3-arylcoumarin ring can promote the inhibition of MAO-B. Compound **78** has the trifluoromethyl group (IC_50_ = 0.056 µM) at the *para* position of the 3-benzene ring, which is the most active compound in this group. The results further confirm that 3-phenylcoumarin is an ideal framework for constructing effective small molecule MAO-B inhibitors.

### 3.4. Anti-inflammatory

Luciana M. Kabeya's group has used 20 hydroxylated and acetoxylated 3-phenylcoumarin derivatives as the inhibitors of oxidative metabolism of neutrophils stimulated by immune complexes. At the same time, as type III possible modulators of inflammatory tissue damage found in the allergic response were determined. The hydroxylated and acetoxylated derivatives **79** and **80** are the most effective inhibitors, and their IC_50_ values in CL-LUC and CL-LUM tests were significantly lower than those of the standard compound quercetin^11^.

Xanthine oxidase (XO) catalyses the conversion of xanthine to uric acid. Excessive uric acid in the human body can lead to diseases such as inflammation and gouty arthritis. Matos et al.^12^ designed a series of 3-arylcoumarin derivatives as a xanthine oxidase inhibitor. Compound **81** (4′-methoxyphenyl-6-nitrocoumarin) proved to be the best XO non-competitive inhibitor in this class of compounds, providing a basis for further design of non-purine XO inhibitors.

Micássio et al. reported the effects of 10 aryl coumarin derivatives on the regulation of superoxide anion and total ROS production by human neutrophils stimulated by immune complexes. Most compounds did not have a significant effect at the beginning of the neutrophil ROS generation process, because they did not significantly inhibit NADPH oxidase activity. The most effective CL-Lum inhibitors, compounds **82** and **70a**, had stronger inhibitory effects on myeloperoxidase and stronger ability to scavenge hypochlorous acid but did not affect NADPH-oxidase activity. Their IC_50_ values were significantly lower than Quercetin^43^. Later, such compounds reduce neutrophil oxygen free radicals and total ROS levels by eliminating oxidant substances and inhibiting myeloperoxidase activity instead of NADPH oxidase complex activity.

Nitric oxide (NO) is one of the inflammatory mediators in the human body and is secreted by activated immune cells (such as macrophages). High levels of NO under chronic inflammatory may cause various pathological conditions^13^. In order to find new compounds with anti-inflammatory activity, Pu et al. used the Perkin reaction to synthesise a series of 3-arylcoumarin compounds. Activity evaluation was performed in macrophages of lipopolysaccharide-inactivated mice, 6,8-dichloro-3–(2-methoxyphenyl) coumarin (compound **83**) and 6-bromo-8-methoxy-3–(3-methoxyphenyl) coumarin (compound **84**) with IC_50_ of 8.5 µM and 6.9 µM, respectively. And Compounds **83** and **84** can be used as a potential anti-inflammatory lead compound for further study.

### 3.5. Anti-PD activity

Parkinson’s disease (PD) is associated with degeneration of dopaminergic substantia nigra striatum neurons. PD, also known as paralysis agitans, is a common chronic progressive dyskinesia of the central nervous system. The main clinical symptoms were tremors at rest, bradykinesia (typical swaying gait when walking, difficulty in starting activities, and inability to stop or change direction in time once started), rigidity and postural and motor imbalance. A few patients have memory impairment and dementia^112^. Selective MAO-A inhibitors are used to treat neurological disorders such as anxiety and depression. Selective and irreversible MAO-B inhibitors have been shown to be useful in the treatment of Parkinson's disease (PD) and Alzheimer’s disease (AD). Therefore, inhibition of MAO-B activity can not only prolong the residence time of dopamine in the brain but also indirectly protect neurons. Previous studies have suggested that MAOI-B is neuroprotective in PD by slowing disease progression^113^. All these problems have led to an intensive search for the new MAOIs.

Ferino et al.^114^ synthesised a series of 3-arylcoumarin derivatives through the Perkin condensation. The activities of hMAO isomers were measured, and most of the compounds showed selectivity to hMAO-B isozymes with high affinity in the range of micron and nano-molar concentrations. Compound **81** proved to be the most active in the series. The properties of the interaction between the compound and hMAO-B were determined by the molecular docking method, which rationalised the structure-activity relationship of the synthetic compound. The results encourage the group to further explore the potential of this family of compounds as candidate medicines for Parkinson’s disease.

Due to the complex aetiology of Parkinson’s disease, Sashidhara et al.^115^ turned to designing multitarget-directed ligands (MTDLs) that can interact with multiple targets at present. So, a multifunctional 3-arylcoumarin-tetracyclic tacrine derivative for the treatment of Parkinson’s disease (PD) was designed and synthesised in this work. Transgenic C. elegans models were used to assess the effects of these compounds on PD. Compound **85** was identified as inhibiting the aggregation of α-synuclein protein, increasing the content of dopamine, and showing good antioxidant properties. It can be used as a new and interesting structure to treat Parkinson’s disease.

### 3.6. Anti-AD activity

Alzheimer’s disease (AD) is a condition characterised by progressive cognitive impairment and memory impairment, which is named neurodegeneration. The clinical manifestations include a general and lasting decline in memory, judgement, calculation, abstract thinking and language, emotional and behavioural disorders, loss of working ability and independent living ability^116^. At present, the aetiology of AD is not clear, generally considered to involve genetic factors and environmental factors. The cholinergic hypothesis suggests that cognitive and memory decline concerned with AD is caused by a selective decline in acetylcholine. There are no therapeutic drugs for the cause of AD, but some drugs can delay the progress of AD and improve the memory and cognitive function of AD patients. The drugs applied to treat AD are acetylcholinesterase inhibitors and NMDA receptor antagonists.1 It had been reported that MAO was an important target for the treatment of AD. MAO, exists as two isozymes, selective MAO-B inhibitors can be used to treat neurodegeneration diseases, such as AD.

In this review, a series of 6-substituted 3-arylcoumarin derivatives were designed and synthesised, and as a selective AChE/MAO-B double inhibitor evaluated in biology. Most of the compounds could inhibit hAChE and hMAO-B effectively and selectively. The activities of all target compounds to ChEs were better than that of the precursor compound, which indicated that the introduction of the amino side chain could enhance the inhibition ability of the compounds. Compounds **86** and **87** showed higher inhibition activity of hMAO-B, stronger hAChE inhibitory activity, and notable inhibition of Aβ42 aggregation^117^.

Hu et al.^116^ designed and synthesised a series of 3–(4-aminophenyl)-coumarin derivatives, which were characterised and evaluated *in vitro* and in vivo. Compound **88a** (IC_50_ = 0.091 ± 0.011 µM) showed the highest effective AChE inhibitory activity, which was slightly weaker than that of the positive control donepezil (IC_50_ = 0.012 ± 0.001 µM). The inhibitory activity of compound **88b** on BuChE was 5 times that of donepezil (IC_50_ = 0.559 ± 0.017 µM), which was the strongest among the compounds. Compound **88a** can be used as attractiveness in the development of medicine design for the treatment or prevention of AD^72^.

### 3.7. Anti-diabetic activity of 3-arylcoumarin derivatives

Diabetes mellitus (DM) is a group of metabolic diseases characterised by the long-term increase in blood glucose levels due to the deficiency of insulin secretion. Its main harm comes from complications, which will lead to high mortality and disability rate. It has been documented that 3-arylcoumarin derivatives significantly reduce blood glucose levels in genetically diabetic mice^118^. 3-arylcoumarin derivatives have antioxidant properties. The characteristics of an antioxidant compound can protect oxidative stress, relieve symptoms and improve organ function in the early stage of diabetic nephropathy. And reduce the neurotoxicity related to diabetes by normalising the myelin sheath of nerve fibres and pain behaviours^119^.

According to Hu et al.^120^ a variety of substituted 3-arylcoumarin derivatives were synthesised by microwave irradiation. Those compounds were screened for α-glucosidase inhibition, antioxidant and advanced glycation end (AGEs) product formation inhibition. Compound **89** has the same activity as the positive control glibenclamide, providing a potential drug target for the development of treatment or prevention of diabetes and diabetic complications. It is worth noting that, compared with traditional methods, this synthesis method has the advantages of environmental friendliness, economy and convenience, simple separation and purification process, fewer by-products and high yield.

### 3.8. Antibacterial activity

Goth first described the antibacterial properties of coumarin and its derivatives in 1945. In recent years, the abuse of antibiotics brings about the phenomenon of multiple drug resistance. Therefore, it is urgent to find new antimicrobial agents. Matos et al. synthesised a series of different substituted amino/nitro 3-arylcoumarins. A series of substituents (methoxy, bromo, nitro, amino and methyl groups) were linked to the nucleus of 3-arylcoumarin in order to search for antibacterial activity and selective structure. The results exhibited that 8 of the 3-arylcoumarin derivatives had inhibitory activity against S. aureus. MIC analysis showed that different compounds had different inhibitory effects on S. aureus due to different kinds and positions of substituents. The activity of 3–(3′-methylphenyl)-6-nitrocoumarin (Compound **90**, MIC = 8 µg/mL) was the best. It has been shown that the nitro substituent at the C6 site is essential for the antibacterial activity of these compounds, but the presence of amino groups decreases the activity of the compounds^119^.

Antibiotic resistance is one of the insurmountable problems of this century. Studies have found that this resistance is also exchanged with oxidative stress, which may help to select drug-resistant bacteria strains. According to Pisano et al.^121^ a series of hydroxy-3-arylcoumarin derivatives with flavonoid structure characteristics were studied, and their antibacterial and antioxidative activities against different bacterial strains were evaluated. Compounds showed high selectivity against Gram-positive bacteria compared to Gram-negative bacteria. Compound **91** showed the best activity against Bacillus cereus and Staphylococcus aureus with minimum inhibitory concentrations of 11 µg/mL. Based on the structure-activity relationship, the number and position of the hydroxyl groups on the coumarin mother's core and 3-phenyl group may affect the antibacterial properties of the compound.

Bacteroides fragilis is an anaerobic bacterium that naturally exists in the human colonic flora. Ugurel et al.^6^ based on the structure of B. Fragile D-lactate dehydrogenase (BFD-LDH), a drug design was performed to test the inhibitory ability and structural relationship of coumarin derivatives on D-LDH. In 3-arylcoumarins, compounds **70a** and **70b** had the highest inhibitory activity, with IC_50_ values of 0.47 ± 0.002 and 0.05 ± 0.001 µM, respectively. And the reversible non-competitive inhibition of the BFD-LDH inhibition mechanism.

Tenacbaculum Maritimum is the pathogen of refractory bacterial disease in marine fish, and it is a Gram-negative filamentous bacterium. Therefore, Serra et al.^122^ synthesised 3-arylcoumarin derivative as a new type of antibacterial compound, and its antibacterial activity against bacteria was evaluated. Preliminary results indicate that compound **91** showed the best activity with a zone of inhibition of 38 mm. It can be further optimised as a new scaffold for building effective antibacterial drugs.

### 3.9. Tyrosinase inhibitory activity

Melanogenesis is a physiological pathway for melanin formation. Tyrosinase catalyses the first step of this process and downregulating its activity is responsible for inhibiting melanin production. Finding molecules that control hyperpigmentation is a hot topic in health and cosmetics. Francesca et al.^19^ synthesised a series of 3-arylcoumarin derivatives and tested for their inhibitory activity on mushroom tyrosinase and murine melanoma B16F10 cells. Compared with the positive control kojic acid (IC_50_ = 17.91 ± 0.98 µM), compound **92** showed a higher tyrosinase inhibitory activity (IC_50_ = 1.05 ± 0.056 µM).

In order to find out the structural characteristics of tyrosinase inhibitory activity, Matos et al.^123^ designed and synthesised a series of new 3-arylcoumarin compounds with different substituents. Its inhibitory activity against tyrosinase was also evaluated *in vitro*. Compound **93** (IC_50_ = 215 µM) was found to be the most active compound in the series. As expected, the more hydroxy substituents on the coumarin core, the stronger the compound's tyrosinase inhibitory activity.

Matos et al.^124^ synthesised a series of selected 3-aryl and 3-heteroarylcoumarin derivatives in 2015 and evaluated their inhibitory activity on mushroom tyrosinase. Compound **92** (IC_50_ = 1.05 ± 0.056 µM) was the most active competitive inhibitor in this series of compounds. It was 100 times more active than the positive control kojic acid. Matos et al. concluded that the presence of dihydroxy groups at positions 5 and 7 of the coumarin backbone could increase the inhibitory activity. This result encourages researchers to continue their efforts to optimise the chemical structure of compound **92** with the aim of obtaining better pharmacological activity.

### 3.10. Anti-insect activity

According to Natalia et al.^20^ compound **94** was found to be effective against parasitic upper flagellate and flagellates in an *in vitro* study of trypan killing capacity, and its activity on the upper flagellate form was higher than that of the positive control nitrofuraniox 13 times. The presence of catechol groups at the 7,8-position of the coumarin core has been shown to be critical for trypanosomal activity. The production of free hexane in the form of coumarins after incubation with coumarin suggests that cytotoxic mechanisms may occur through oxidative stress on parasites.

### 3.11. Adenosine affinity

Adenosine receptors are GPCRs (G-protein-coupled receptors) and have been described as potential therapeutic targets for a wide range of pathologies. In the past few years, people have begun to look for agonist and antagonist ligands that bind to a single AR subtype. Most AR antagonists are promising lead compounds for the treatment of certain diseases, such as inflammation, Parkinson’s syndrome, and Alzheimer’s disease. In particular, the A_3_AR subtype has been used as a potential therapeutic target for the prevention of ischaemic heart and brain damage. Nowadays, due to its higher density expression in the liver, lung, neutrophils, macrophages and glial cells, it can be used in inflammation and immunosuppression to play an essential role. Therefore, A_3_AR ligands have therapeutic potential for diseases such as cancer, myocardial ischaemia, and hepatitis^125^^,^^126^.

Matos et al.^125^ a series of 3-arylcoumarin derivatives were reported in 2013, and those compounds *in vitro* selectivity and binding activity to human adenosine receptor (AR) were evaluated. The effects of different substituents on the binding affinity at the 3′, 4′, and C6 positions of the 3-arylcoumarin skeleton. None of the derivatives had detectable binding to hA2A and hA2BAR. Among them, compound **74a** had the most significant activity and the best affinity for hA3AR (Ki = 2680 nm) and had significant selectivity for this isoform.

Matos et al.^126^ selected different 8-substituted-3-arylcoumarins as ligands for adenosine receptors for research in 2019 and performed radioligand binding analysis. Among the synthesised compounds, compound **95** showed the highest potency and selectivity as an A_3_ receptor antagonist (Ki = 258 nM) and can be used as an inspiration for the development of new coumarin-type powerful and selective A_3_ antagonists. In particular, 8-substituted compounds have different affinity and selectivity and are a promising class of adenosine receptor antagonists.

### 3.12. Other activities

Quezada et al.^14^ synthesised a series of 6-halo-3-hydroxyphenylcoumarins hybrids (resveratrol-coumarins hybrid) by virtue of Perkin reaction with high yields. The vasodilating activity of these compounds and the thrombin-induced human platelet aggregation was evaluated. It was found that the antiplatelet activity of compound **96** (IC_50_ = 6.41 ± 2.15 µM) inhibited platelet aggregation more effectively than t-RESV when thrombin (0.25 U/mL) was used as a stimulant. There are different halogen substitutions at the 6-position carbon of the coumarin nucleus, and derivatives with significantly higher pharmacological activity than t-RESV can be obtained.

With the surge in the prevalence of allergic diseases, there is increasing interest in inhibitors of such diseases. And in recent years, 3-arylcoumarin derivatives have been considered as lead compounds with the potential to treat allergic diseases. Therefore, Marcela et al.^127^ screened a group of 3-arylcoumarins as potential inhibitors of mast cell degranulation as new antiallergic drugs. The compound **97** (IC_50_ = 10.5 ± 1.8 uM) was a more effective mast cell degranulation inhibitor than the positive control ketotifen.

Andrade et al.^3^ a series of 3-arylcoumarin derivatives containing free and acetylated hydroxyl groups have been studied for their antioxidant and immunomodulatory properties, with the goal of developing drugs that can be used to treat inflammatory diseases in humans. The role of synthetic 3-phenylcoumarin derivatives in Fcγ receptor (FcγR)-mediated human neutrophil oxidative metabolism was evaluated. The 6,7-disubstituted compounds down-regulated Fcγ receptor-mediated oxidative metabolism in neutrophils more than the 5,7-disubstituted compounds. And the hydroxyl compounds down-regulated neutrophils function better than the acetyl compounds. Among them, compound **82** was the most active, not only regulating neutrophil oxidative metabolism but also down-regulating immune complex-stimulated cytophagy and elastase degranulation.

Recently, tropical Taylor disease has developed resistance to the commonly used antimalarial drug paracetamol, so new alternative treatments have to be found. Theileria annulata enolase plays an important role in the glucose metabolism of worms and is considered being a potentially useful pharmaceutical protein. Yakarsonmez et al.^32^ with Theileria annulata enolase (TaENO) as the research target, a series of 3-arylcoumarin derivatives were synthesised as TaENO inhibitors using a structure-based drug design strategy. The compound **70b** (IC_50_ = 10.863 µM) had the strongest inhibitory effect. Kinetic results showed that compound **66b** was a non-competitive enzyme inhibitor. The results will help further structure-based drug design research.

Bakhchinian et al.^128^ found that 3-arylcoumarins have some binding capacity to oestrogen receptors but have poor selectivity for human α and β oestrogen receptors. In the past two years, the 3-arylcoumarin derivatives studied by Bakhchinian et al. showed a good binding ability to oestrogen receptors.

Niinivehmas et al.^129^ synthesised a series of 3-arylcoumarin compounds with polar substituents to block the synthesis of 17-b-hydroxysteroid dehydrogenase 1 (HSD1). The results demonstrate that 3-phenylcoumarin is a suitable non-steroidal backbone for constructing effective and selective HSD1 inhibitors.

## 4．Conclusions and perspective

The present papers reviewed, various aspects of 3-arylcoumarin derivatives, including their natural product sources, biological activities, and chemical synthesis. 3-arylcoumarin is a simple molecule and its derivatives have been documented in the treatment of various disorders. As discussed in this article, the MAO-B inhibitory activity of 3-arylcoumarin is optimum. The reported assay techniques suggested that compounds **41** (IC_50_ = 0.3 × 10^−3 ^µM) and **42** (IC_50_ = 0.80 × 10^−3 ^µM) and all derivatives with IC_50_ values range from micro to nano molar^107^. These findings encourage the pharmacist optimise the pharmacological profile of a structural moiety as an important scaffold for the potential treatment of Parkinson’s disease or Alzheimer’s disease. And in developing green synthetic strategies, science mainly concentrates on avoiding environmentally non-compatible reagents, solid-phase synthesis, modification of synthetic routes to decrease the number of steps and increase overall yield, usage of newer catalysts and simplification of classical procedures of reaction. Such as the microwave radiation heating method is environmentally friendly and economical, with low by-products and high yield. Although remarkable progress and breakthroughs have been made in the research of biological activity and chemical synthesis of 3-arylcoumarins, there is still a long way to go. Improving the properties of the compounds is paramount. For example, the existing studies lack plentiful papers aiming at the 3-arylcoumarin structure to support` as a multi-target therapy for neurodegenerative diseases. The admixture or aggregation with 3-phenylcoumarin or more pharmacodynamic groups would show a dual mode of action with enhanced biological activity^24^^,^^25^^,^^115^^,^^117^. In terms of synthesis, conventional toxic substances such as transition metals were abandoned as catalysts in search of simple and convenient synthetic routes. In conclusion, despite the prominent new results obtained in the last decades, the design of potent and selective 3-aryl coumarins confers favourable properties, especially for multiple pharmacological activities for clinical applications. This remains a formidable challenge for researchers. So, 3-arylcoumarins will probably remain on the scene for some time, even if no new 3-arylcoumarins are advanced to clinical trials for several years.
